# Common Bile Duct Stone Formed around a Migrated Clip: An Unexpected Complication of Laparoscopic Cholecystectomy

**DOI:** 10.1155/2018/5892143

**Published:** 2018-05-13

**Authors:** Anas M. Hussameddin, Iba Ibrahim AlFawaz, Reema Fahad AlOtaibi

**Affiliations:** ^1^Department of Internal Medicine, King Fahd University Hospital, Khobar, Saudi Arabia; ^2^College of Medicine, Imam Abdulrahman Bin Faisal University, King Fahd University Hospital, Dammam, Saudi Arabia

## Abstract

Surgical clip migration into the common bile duct with subsequent stone formation is a rare complication following laparoscopic cholecystectomy. Very few cases have been reported in the literature. We report a case of bile duct stone formation around a migrated surgical clip 16 years after laparoscopic cholecystectomy. The patient presented with right upper quadrant pain, fever, and chills for one week. Investigation with abdominal ultrasound showed dilatation of the common bile duct and moderate dilatation of the intrahepatic bile ducts. The diagnosis was confirmed by endoscopic retrograde cholangiopancreatography and the patient was managed successfully with sphincterotomy and stone extraction. The exact mechanism of clip migration is not fully understood. Presenting symptoms are similar to non-clip-induced choledocholithiasis. Time of presentation can vary significantly with an average of 26 months. Most cases reported in the literature required surgical intervention. Clip migration should be considered in the differential diagnosis of postcholecystectomy biliary colic and cholangitis. Management with endoscopic retrograde cholangiopancreatography is the treatment of choice.

## 1. Introduction

Laparoscopic cholecystectomy has been the treatment of choice for symptomatic gallstone disease for the past two decades. It carries a high rate of success with fewer complications and shorter hospital stay compared to open cholecystectomy [1].

Surgical clip migration is an uncommon complication of laparoscopic cholecystectomy, which may occur days to years following surgery. Migration of the clips into the common bile duct can lead to stone formation and obstruction. It should be considered in the differential diagnosis of postcholecystectomy biliary colic and cholangitis.

## 2. Case Report

A 70-year-old male presented with recurrent right upper quadrant pain, chills, and fever for one-week duration. He was previously treated with laparoscopic cholecystectomy for symptomatic gallstones at our hospital. After cholecystectomy, the cystic duct was ligated with endoclips. The postoperative period was uneventful. Sixteen years later, the patient presented with a picture suggestive of ascending cholangitis. Upon recent presentation, physical examination revealed jaundice in the sclera with tenderness at the right upper quadrant of the abdomen on deep palpation. No mass was palpable. His vital signs revealed a temperature of 38.7°C, heart rate of 107 beats per minute, blood pressure of 157/67 mmHg, and respiratory rate of 20 breaths per minute.

*Laboratory Tests*. Total bilirubin was 94 *μ*mol/L, mostly direct bilirubin (normal range: 2–18 *μ*mol/L), AST was 436 U/L (normal up to 40 U/L), ALT was 369 U/L (normal up to 35 U/L), alkaline phosphatase was 176 U/L (normal 70–120 U/L), and GGT was 585 U/L (normal up to 60 U/L).

Abdominal ultrasound showed dilated common bile duct (11 mm), with intrahepatic biliary duct dilatation.

**Figure 1:**
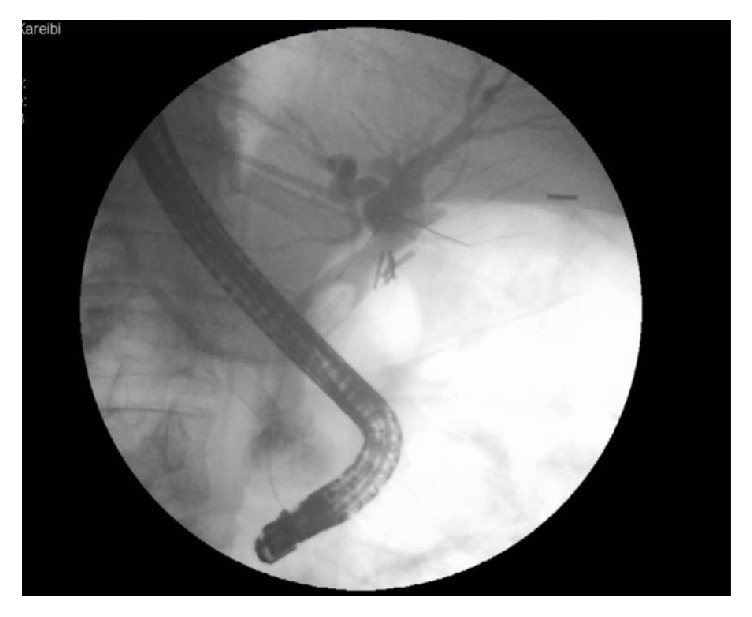
ERCP showing a large filling defect in a dilated common bile duct with central opacity.

An endoscopic retrograde cholangiopancreatography (ERCP) was performed which showed dilated common bile duct with big filling defect and central radiopaque density (Figure 1).

Sphincterotomy was performed with successful complete stone extraction by a balloon.

## 3. Discussion

Clip migration is an infrequent complication after laparoscopic cholecystectomy, which may cause common bile duct obstruction secondary to stone formation.

The exact mechanism of clip migration is not fully understood.

Kitamura et al. proposed that clip migration happens when the surrounding structures, such as the liver, press the clipped cystic duct, which is then inverted into the lumen of the common bile duct. Eventually, the inverted cystic duct becomes necrotic and clips fall into the common bile duct [2].

Other suggested methods reported by V. H. Chong and C. F. Chong include incorrect clip placement resulting in bile duct injuries, placement of too many clips, and placing clips in the biliary tree [3].

The time of clinical presentation with postcholecystectomy clip migration has been reported to vary from 11 days to 20 years with a median of 26 months. Symptoms of clip-induced gallstones are similar to the common causes of gallstones. Common presentations of postcholecystectomy clip migration were obstructive jaundice (37.7%), cholangitis (27.5%), biliary colic (18.8%), and acute pancreatitis (8.7%) [3].

Postcholecystectomy clip migration is one of the differential diagnoses of patients with biliary colic or cholangitis after cholecystectomy.

Most case reports revealed that the stone formed around migrated clips required surgical treatment because of its large size. However, in some cases, proper sphincterotomy by ERCP with basket and balloon extraction can be effective [4].

Surgical clip migration can be partly prevented by correct placement and use of a minimal number of clips. However, even correctly placed clips might migrate into the biliary tract if local ischemia or infectious processes occur, consequently leading to the formation of gallstones around the migrated clip [5].

ElGeidie proposed a new technique of clipless laparoscopic cholecystectomy, which is feasible, practical, and safe. It was suggested that the use of ligation is associated with a reduced risk of postoperative complications [6].

## References

[1] Holohan T. V. (1991). Laparoscopic cholecystectomy.

[2] Kitamura K., Yamaguchi T., Nakatani H. (1995). Why do cystic duct clips migrate into the common bile duct?.

[3] Chong V. H., Chong C. F. (2010). Biliary complications secondary to post-cholecystectomy clip migration: a review of 69 cases.

[4] Gonzalez F. J., Dominguez E., Lede A., Jose P., Miguel P. (2011). Migration of vessel clip into the common bile duct and late formation of choledocholithiasis after laparoscopic cholecystectomy.

[5] Cetta F., Baldi C., Lombardo F., Monti L., Stefani P., Nuzzo G. (1997). Migration of metallic clips used during laparoscopic cholecystectomy and formation of gallstones around them: surgical implications from a prospective study.

[6] ElGeidie A. A. (2012). New Technique of Clipless Laparoscopic Cholecystectomy.

